# Consumption: How to Prevent It, and How to Live with It

**Published:** 1892-03

**Authors:** 


Consumption: How to Prevent it and How to Live With it; Its Nature, its Causes, its Prevention, and the Mode of Life, Climate, Exercise, Food, Clothing necessary for its Cure. By U. S. Davis, Jr., A. M., M. D., Professor of the Principles and Practice of Medicine, Chicago Medical College, etc., etc., Philadelphia, 1231 Filbert Street, and London. F. A. Davis, Publisher. 1891. Price, 75 cents, net.

This book has been written for the intelligent laity, but may be studied by the physician to good advantage. The author gives extensive hints on the nature of consumption, and on the means of preventing infection and predisposition. He speaks of the prevention of consumption and the hygiene for consumptives, and finally gives some general hints on the treatment of consumption, as far as a non-professional can treat himself. It is good reading to place in the hands of any individual having weak lungs. We spent a pleasant afternoon perusing its pages. W. S.

